# Supplementary material for: HIVprotI: an integrated web based platform for prediction and design of HIV proteins inhibitors
Source: J Cheminform. 2018 Mar 9;10:12. doi: 10.1186/s13321-018-0266-y (PMC5845081; doi:10.1186/s13321-018-0266-y)
Supplement: Supplementary file 2 — Additional file 2. Source code of HIVProtI web server. [file 13321_2018_266_MOESM2_ESM.zip › HIVprotI_Source-code/hivprotI/jsme/JSME_callback_and_star.html]

Structure modified and atom mark demo


# Structure modified and atom mark demo

Draw a structure, click on the star icon of the editor and click on one or more atoms

Clear log
  
After structure changed event:
  
 
  

|  |  |  |  |
| --- | --- | --- | --- |
| Import predefined chemical structure to applet: | | | |
| Read MOL | Read JME String | Read Multipart Structure | Read reaction |

  

|  |  |
| --- | --- |
| Set options: |  |
| reaction,nostar | Set options |
